# Lipids and lipoproteins and inflammatory markers in patients with chronic apical periodontitis

**DOI:** 10.1186/s12944-015-0156-5

**Published:** 2015-12-14

**Authors:** Aleksandra Kimak, Małgorzata Strycharz-Dudziak, Teresa Bachanek, Elżbieta Kimak

**Affiliations:** Department of Conservative Dentistry with Endodontic, Medical University, Karmelicka 7, 20-081 Lublin, Poland; Department of Laboratory Diagnostics, Medical University, Street Chodźki 1, 20-093 Lublin, Poland

**Keywords:** Lipids, lipoproteins, TNF-α, IL-6, hsCRP, LpPLA2, Chronic apical periodontitis

## Abstract

**Background:**

Since chronic apical periodontitis (CAP) appears to be a risk factor for coronary heart disease, the aim of the study was to determine the relationship between the size of CAP lesion and inflammatory markers (hsCRP, IL-6, TNF-α), as well as lipids and lipoproteins (LpPLA2, apoAI, apoB level) in blood serum of patients with CAP.

**Methods:**

The patients studied (*n* = 43) were divided into groups: patients under 50 and over 50 years of age, and a separate subgroup of the oldest age with the largest size of CAP lesions. Apolipoprotein AI (apoAI) above 150 mg/dL and below 150 mg/dL was used as an important criterion for the division of patients into groups. The CAP lesion size was measured using the Kodak digital imaging system software. The control group consisted of clinically healthy volunteers (*n* = 20) without CAP. Lipids were measured on a Siemens analyzer (Germany), apoAI, apoB, hsCRP levels were determined by immunonephelometric method, using the Health Care Diagnostic Product (Siemens GmbH, Germany), and IL-6, TNF-α and LpPLAG7 assay kits (ELISA, R&D Systems) were used.

**Results:**

The findings suggested that in patients with CAP and their age increase, the CAP lesion size, the concentration of inflammatory markers and LpPLA2 mass increased. Correlations between the CAP lesion size and LpPLA2 mass and between the CAP lesion size and TG level in patients with apoAI 150 ≤ mg/dL showed increase TG in atherogenic apoB-containing triglyceride-rich lipoprotein and TC in cholesterol-rich lipoprotein. The patients with a low apoAI and high LpPLA2 level can have a higher risk of odontogenic disease and progression of atherosclerosis and coronary heart disease.

**Conclusion:**

We have found a positive correlation between apoAI level and the CAP lesion size and a negative correlation between LpPLA2 level and the CAP lesion size. The results suggest that apoAI and LpPLA2 in HDL particles have antiinflammatory action and together can limit the CAP lesion size in patient with a higher apoAI level. The literature data on the distribution of lipoprotein particles in subjects are still insufficient, so this problem requires further studies.

## Background

Atherosclerosis is considered as an inflammation-mediated process initiating the focal accumulation of lipids into the arterial intima and driven by complex interactions between leukocytes, platelets and cells of the vessel wall [1]. So, the role of serum lipids and lipoproteins is significant in this inflammatory process [2, 3]. Inflammation occurs as a result of exposure of tissues and organs to harmful stimuli such as microbial pathogens, irritants, or toxic cellular components. Inflammation processes involve the major cells of the immune system [4]. Interleukin-6 (IL-6) is a cytokine, which plays an important role in many chronic inflammatory diseases [5]. The author’s previous findings had shown that serum and gingival crevicular fluid (GCF) proinflammatory cytokines such as TNF-α, IL-1b, and IL-6 levels may play an important role in association with a periodontal disease and hyperlipidemia [3]. The release of IL-6 from several cell types, including smooth muscle cells was induced by TNF-α and IL-1. The effects of IL-6 and TNF-α on lipid metabolism may develop endothelial generation of nitric oxide in consequence of which are raising circulating concentrations of non-esterified fatty acids [6]. The effect of IL-6 on platelets, fibrinogen concentrations, and coagulation and of TNF-α on expression of plasminogen activator inhibitor by hepatocytes, endothelial cells, and adipose tissue may lead to a procoagulant state in such subjects [7]. Therefore, reduction of the inflammatory mediators such as TNF-α and IL-6 (serum and/or GCF), which are also associated with both hyperlipidemia and periodontitis may provide a further contribution to a two-way relationship between periodontitis and hyperlipidemia [3, 8]. Oxidative stress and atherogenic dyslipidaemia have synergistic impact on atherosclerosis and cardiovascular diseases [9, 10]. In this context, lipoprotein associated phospholipase A2 (Lp- PLA2), an enzyme that has been shown to be a risk factor for cardiovascular disease (CVD) and is believed to be an independent CVD risk factor that is also involved in degradation of the phospholipid mediator platelet-activating factor (PAF) [11–13] was significantly reduced by treatment of periodontitis [9, 14]. Unfortunately, the mechanism by which these cytokines increase the serum lipid and lipoprotein levels is unknown. Hence, a little information about the relationship between chronic apical periodontitis (CAP) and lipoproteins is available. CAP is defined as a persistent inflammation of tissues adjacent to the apex of the tooth root. It is usually called asymptomatic because it probably does not produce any clinical symptoms, but on radiographs destruction of the periapical tissues with possible resorption of the root and bone is noted. Periapical disease is caused by opportunistic pathogens infecting the dental pulp and periapical tissues therefore the treatment of the disease involves elimination of microorganisms in the course of endodontic treatment. The activity of the microorganisms is associated with host response [15–19].

The aim of the study was to determine the relationship between the size of chronic inflammatory periapical lesions and the inflammatory markers (hsCRP, IL-6, TNF-α level), and lipids, and lipoproteins (LpPLA2, apolipoprotein (apo) AI, apoB concentration) in the blood serum of patients with CAP.

## Methods

### Patients

The study comprised 43 patients admitted to the Department of Conservative Dentistry with Endodontics at the Medical University of Lublin and a private dental practice. The study was conducted in accordance with the guidelines of the Ethics Committee of the Medical University in Lublin, Poland. The patients were in a good health condition. They (*n* = 43) were divided into groups: one up to 50 years of age and the other over 50 years of age. A separate subgroup of the studied patients was that of the oldest age and with the largest CAP lesions size. ApoAI above 150 mg/dL and below 150 mg/dL as an important criterion for the division into groups of patients was used. A periapical X-ray of each tooth with apical inflammatory lesion was taken by using of a sensor holder and the Kodak digital radiography system. The size of the lesions (mm^2^) was measured using the Kodak digital imaging system software. If more than one periapical lesion was found in a patient, the surfaces of all lesions were summed up. All patients were without statin therapy. Patients with CAP did not have periodontal disease and did not receive lipid-lowering drugs, nine patients were taking antihypertensive medications that do not effect on lipids. No patient had diabetes, a liver or kidney disease, cancer, acute inflammation, a stroke and acute cardiovascular events. They did not habitually drink alcohol and did not smoke tobacco. The control group consisted of 20 clinically healthy students and volunteers from the laboratory (9 men and 11 women) aged from 23 to 50 years without CAP. The absolute selection criteria in this group of patients were normal BMI, correct results of routine laboratory parameters, hsCRP, lipids, lipoproteins apoAI and apoB lipid and lipoprotein ratios.

### Sample preparation

Whole blood from the patients was drawn after 14 h of fasting. The blood was taken from a vein in to commercial test tubes. Red blood cells were separated from plasma by centrifugation at 6000 rpm for 15 min at 4 °C (Eppendorf Centrifuge 5810R). Serum was immediately separated and stored in aliquots at 80 °C until use.

### Detection of lipids, lipoproteins, hsCRP

Lipids, lipoproteins, and inflammatory markers were obtained from serum after 14-h overnight fasting. Lipids were measured on a Siemens analyzer (Germany). Clinical parameters are listed in Table 1. Low density lipoprotein cholesterol (LDL-C) was calculated according to the Friedewald formula. Non-high density lipoprotein cholesterol (non-HDL-C) was calculated as total cholesterol (TC) minus HDL-C. Lipoproteins apoAI, apoB, hsCRP were determined by immunonephelometric methods, using the Health Care Diagnostic Product (Siemens GmbH, Germany) on a Dade Behring nephelometer BNII System (Germany) [20].

**Table 1:** Clinical parameters in the control group, in the group of patients ≤ 50 years of age and > 50 years of age and in all patients with chronic apical periodontitis (CAP), median (min- max)

	Control Group	Patients ≤ 50 years of age	Patients > 50 years of age	All patients
	*n* = 20	*n* = 20	*n* = 23	*n* = 43
Age, years	40(32–50)	41(32–50)	63(58–69)*	61(32–69)*
Sex (male, female)	9 M, 11 F	9 M, 11 F	13 M, 10 F	22 M, 21 F
BMI kg/m^2^	23(21–25)	23(20–28)	25(21–38)*	24(20–38)
Hypertension	-	3	6	9
Statin therapy	-	-	-	-

*P* < 0.05-* vs control group

### Detection of IL-6, TNF-α and LpPLAG7

Serum IL-6, TNF-α and LpPLA2G7 assay kits (ELISA, R&D Systems) were used. The kit is a sandwich enzyme immunoassay for *in vitro* quantitative measurement of human IL-6 or TNF-α or LpPLA2G7 of serum. A monoclonal antibody specific for IL-6 or TNF-α or LpPLA2G7 was pre-coated on to a microplate. Standards and samples were pipetted into wells, and any IL-6 or TNF-α or LpPLA2G7 present was bound by immobilized antibody. After washing away any unbound substances, enzyme-linked polyclonal antibody specific for IL-6 or TNF-α or LpPLA2G7 was added to the wells. After an incubation period amplifier solution was added to the wells, and the colour developed in proportion to the amount of IL-6 or TNF-α or LpPLA2G7 bound in the initial stage. The colour development was stopped and the intensity of the color was measured.

### Statistical analysis

The data were expressed as medians and minimum-maximum. Statistical analysis of the results was performed using the non-parametric Kruskal-Wallis (KW) test for comparison of the CAP patient groups and the control group. The relation between the CAP lesion size and the inflammatory markers (hsCRP, IL-6, TNF-α level), and lipids (triglycerides (TG), TC, LDL-C, HDL-C, nonHDL-C) and lipoproteins (LpPLA2, apoAI, apoB concentration), and lipoprotein ratios were examined by Spearman’s correlation analysis. The statistical significance of all variables was established at *p* < 0.05 and the statistical analysis were performed using the STATISTICA software (StatSoft, Krakow, Poland).

### Ethics statement

Written informed consents were obtained from all the participants. The study was approved by the Ethics Committee of the Medical University in Lublin, Poland and conducted according to the principles outlined in the Declaration of Helsinki.

## Results

Table 1 shows the selected clinical parameters in control group, the groups of patients under and over 50 years of age with CAP. The studies showed that in patients under 50 years of age there was no statistically significant increase in the IL-6 and TNF-α concentration as compared to controls, but there was a significant difference between the concentration of hsCRP and LpPLA2 (Table 2). The patients over 50 years of age and their aging showed a significantly large size of CAP lesion and concentration of IL-6 and TNF-α, hsCRP and LpPLA2, and these parameters were the highest in the oldest patients with the largest size of CAP lesion. Table 3 shows the lipid, lipoprotein, and lipid and lipoprotein ratios in the control group and patients under and over 50 years of age and the oldest with the largest size of CAP lesion. These studies confirmed our observations that patients under 50 years of age had the smallest CAP lesion size and did not show statistically significant differences in the concentration of lipids, apoAI, apoB and in the values of lipid and lipoprotein ratios. Moreover, patients over 50 years of age and the oldest with the largest size of CAP lesion had significantly increased lipid and lipoprotein ratios that suggested the risk of atherosclerosis in them. In the studied groups of patients the concentration of lipids, apoAI and apoB did not change as compared to controls, but they showed a significant divergence of the tested parameters from very low to very high, which indicated dyslipidaemia and dyslipoproteinaemia in these patients. Table 4 shows the clinical parameters, the CAP lesion size, markers of inflammation and LpPLA2 mass in the control group and patients with apoAI ≥ 150 mg/dL, with apoAI ≤ 150 mg/dL level and in all patients. The extent of CAP lesion size was similar in all patient groups. In patients with apoAI level ≥ 150 mg/dL the level of LpPLA2 and hsCRP was evaluated, but not the concentration of IL-6 and TNF-α as compared to control. In patients with apoAI ≤ 150 mg/dL the level of all markers of inflammation was significantly elevated in relation to control, and the level of TNF-α and hsCRP was significantly higher in relation to patients with apoAI ≥ 150 mg/dL level. Table 5 shows the concentration of lipids, apoAI, apoB, ratios of lipid and lipoprotein in the control group of patients with apoAI ≥ 150 mg/dL and apoAI ≤ 150 mg/dL level. In patients with apoAI level ≥150 mg/dL no change in lipids, apoAI, apoB concentration and the values of lipid and lipoprotein ratios was shown, but in those with apoAI ≤ 150 mg/dL level significant differences in HDL-C, apoAI and apoB level and of lipid and lipoprotein ratios were shown, which indicated a risk of atherosclerosis and coronary heart diseases.

**Table 2:** Selected clinical parameters, the size of CAP lesion and the concentration of inflammatory markers in the control group, in patients with ≤ 50 years of age and with > 50 years of age, in subgroup of patients with oldest age and the largest the size of CAP lesion, and the group of all patients, the median (min- max)

	Control group	Patients ≤ 50 years of age	Patients >50 years of age	Oldest patients with the largest sCAP	All patients
	*n* = 20	*n* = 20	*n* = 23	*n* = 15	*n* = 43
Age	40(32–50)	41(32–50)	63(58–69)*,***	65(58–69)*,***	61(32–69)*,***
BMI (kg/m^2^ )	23(21–25)	23(20–28)	25(21–38)	26(22–38)*	24(20–38)
sCAP	-	24(4–56)	32(9–120),***	34(23–120),***	32(4–120),***
IL6 (pg/ml)	0.35(0.22–0.55)	0.56(0.13–2.09)	1.17(0.24–4.20)*	1.23(0.40–4.20)*	1.06(0.13–4.20)*
TNF-α (pg/ml)	1.09(0.42–1.55)	1.95(0.31–6.68)	3.96(0.83–8.77)*	4.97(0.92–8.77)*,***	3.37(0.31–8.77)*
LpPLA2(ng/ml)	57(32–93)	153(32–328)*	200(98–328)*	213(172–275)*	188(32–328)*
hsCRP mg/L	0.014(0.017–0.075)	0.268(0.017–0.620)**	0.367(0.010–1.10)**,***	0.424(0.010–1.10**,***	0.338(0.010–1.10)**,***

*P* < 0.05-*; *p* < 0.01-** - vs control group; *p* < 0.05-*** - vs. patients > 50 years of age

sCAP - the size of chronic apical periodontitis lesion

**Table 3:** Concentration of lipid, apoAI, apoB and lipid and lipoprotein ratios in the control group, patients ≤ 50 years of age and > 50 years of age, and in subgroup patients with oldest age and the largest the CAP lesion size and in all patients, the median (min- max)

	Control group	Patients ≤ 50 years of age	Patients >50 years of age	Oldest patients with the largest sCAP	All patients
	*n* = 20	*n* = 20	*n* = 23	*n* = 15	*n* = 43
TG (mmol/L)	1.07(0.70–1.94)	1.27(0.65–3.37)	1.45(0.58–4.26)	1.34(0.58–4.26)	1.33(0.58–4.26)
TC (mmol/L)	4.92(4.35–5.69)	5.12(4.06–7.20)	5.51(3.80-7.40)	5.43(4.25–7.40)	5.38(3.80–7.40)
LDL-C (mmol/L)	3.15(2.20–3.62)	3.60(2.36–5.20)	4.06(2.14–5.23)	3.85(2.69–4.71)	3.78(2.14–5.23)
HDL-C (mmol/L)	1.42(1.13–2.40)	1.37(0.62–2.07)	1.13(0.49–1.99)	1.08(0.49–1.86)	1.16(0.49–2.07)
nHDL-C (mmol/L)	3.62(2.53–4.19)	3.85(2.66–5.82)	4.29(2.64–6.21)	4.06(3.15–6.11)	3.93(2.64–6.21)
apoAI (mg/L)	1680(1500–2030)	1500(790–2350)	1440(950–1820)	1420(1050–1820)	1440(790–2350)
apoB (mg/L)	880(620–1060)	760(520–1340)	970(500–1490)	800(500–1490)	870(500–1490)
TC/HDL-C	3.45(2.17–4.45)	3.67(2.56–6.00)	4.82(2.69–9.0)*	5.01(2.69–9.27)*	4.63(2.56–9.27)
LDL/HDL-C	2.22(1.11–3.72)	2.62(1.35–6.53)	3.57(1.97–9.53)*	3.52(1.54–6.31)*	3.24(1.35–9.53)
TG/HDL-C	1.78(1.0–3.2)	2.13(0.97–8.28)	2.83(0.72–11.8)*	2.83(0.72–7.54)*	2.56(0.72–11.8)
HDL-C/apoAI	0.33(0.32–0.47)	0.35(0.23–0.66)	0.30(0.16–0.50)	0.29(0.0.16–0.48)	0.31(0.16–0.66)
apoAI/apoB	1.89(1.17–3.27)	1.82(1.17–3.17)	1.49(0.70–2.55)*	1.77(0.80–2.45)	1.65(0.70–3.17)

*p* < 0.05-*; sCAP - the size of chronic apical periodontitis lesion

**Table 4:** Clinical parameters, the size of CAP lesion and the concentration of inflammatory markers in the control group and patients with apoAI ≥ 150 mg/dl and apoAI ≤ 150 mg/dl and all patients with CAP, median (min- max)

	Control group	Patients with apoAI ≥ 150 mg/dl	Patients with apoAI ≤ 150 mg/dl	All patients
	*n* = 20	*n* = 18	*n* = 25	*n* = 43
Age	40(32–50)	55(32–69)*	61(37–69)*	61(32–69)
BMI (kg/m^2^ )	23(21–25)	23(20–28)	25(21–38)	24(20–38)
sCAP (mm^2^)	-	33(9.4–120)	32(4–50)	32(4–120)
IL6 (pg/ml)	0.35(0.22–0.55)	0.55(0.220–3.15)	1.14(0.13–4.20)*	1.06(0.13–4.20)*
TNF-α (pg/ml)	1.09(0.42–1.55)	1.21(0.31–8.77)	3.55(0.42–1.24)*,***	3.37(0.31–8.77)*
LpPLA2(ng/ml)	57(32–93)	157(32–327)*	202(80–328)*	188(32–328)*
hsCRP mg/L	0.014(0.017–0.075)	0.155(0.010–0.789)**	0.378(0.010–1.10)**,***	0.338(0.01–1.10)**,***

*p* < 0.05-* ; *p* < 0.01-** - vs control group; *p* < 0.05-*** - vs patients with apoAI ≥ 150 mg/dl

sCAP - the size of chronic apical periodontitis lesion

**Table 5:** Lipids, apoAI, apoB level and lipid and lipoprotein ratios in the control group and patients with apoAI ≥150 mg/dL, apoAI ≤150 mg/dL and all patients with CAP, median (min- max)

	Control group	Patients with apoAI ≥150 mg/dl	Patients with apoAI ≤ 150 mg/dl	All patients
	*n* = 20	*n* = 18	*n* = 25	*n* = 43
TG (mmol/L)	1.07(0.70–1.94)	1.27(0.58–1.84)	1.45(0.65–4.26)	1.33(0.58–4.26)
TC (mmol/L)	4.92(4.35–5.69)	5.49(4.19–7.20)	5.28(3.80–7.40)	5.38(3.80–7.40)
LDL-C (mmol/L)	3.15(2.20–3.62)	4.04(2.20–4.71)	3.72(2.14–5.23)	3.78(2.14–5.23)
HDL-C (mmol/L)	1.42(1.13–2.40)	1.52(1.19–2.07)	1.08(0.49–1.50)*,***	1.16(0.49–2.07)
nHDL-C (mmol/L)	3.62(2.53–4.19)	4.06(2.53–5.23)	4.35-(2.64–6.21)	3.93(2.64–6.21)
apoAI (g/L)	1680(1500–2030)	1740(1500–2350)	1340(790–1480)*,***	1440(790–2350)
apoB (g/L)	880(620–1060)	760(520–1280)	86(0500–1490)	870(500–1490)
TC/HDL-C	3.45(2.17–4.45)	3.58(2.56–4.82)	4.92(2.68–9.27)*	4.63(2.56–9.27)*
LDL/HDL-C	2.22(1.11–3.72)	2.64(1.35–3.54)	3.50(1.80–9.53)*	3.24(1.35–9.53)*
TG/HDL-C	1.72(1.0–3.2)	1.91(0.72–3.26)	3.09(1.07–11.82)*	2.56(0.72–11.82)*
HDL-C/apoAI	0.33(0.32–0.47)	0.33(0.27–0.51)	0.31(0.16–0.66)	0.31(0.16–0.66)
apoAI/apoB	1.89(1.17–3.27)	2.28(1.36–3.27)	1.56(0.70–2.55)*	1.65(0.70–3.17)

*p* < 0.05-*- vs control group

*p* < 0.05-*** - vs apoAI ≥ 150 mg/dl

In all the tested patients a significant positive correlation was shown between:

the concentration of TNF- α and IL-6 level (R = 0.471, p = 0.009), that of TNF - α and hsCRP level (R = 0.608, *p* = 0.00004), that of TNF - α and the age (r = 0.460, p = 0.013 ); the concentration of IL-6 and hsCRP level (R = 0.403, *p* = 0.025), that of IL-6 and the age (r = 0.590, *p* = 0.0007); the concentration of LpPLA2 and TG level (R = 0.537, *p* = 0.0005), that of LpPLA2 and TC level (R = 0.563, *p* = 0.0002), that of LpPLA2 and LDL-C level (R = 0.655, *p* = 0.00008), that of LpPLA2 and nonHDL-C level (R = 0.701 *p* = 0.000001), that of LpPLA2 and TC/HDL-C ratio (R = 0.414, 0.009), that of LpPLA2 and TC/HDL-C ratio (R = 0.418, *p* = 0.009), that of LpPLA2 and LDL-C/HDL-C ratio (R = 0.430, *p* = 0.006) that of LpPLa2 and TG/HDL-C ratio (R = 0.406, *p* = 0.011), that of LpPLA2 and the age (R = LpPLA2, *p* = 0.013).

In the group of patients under 50 years of age we found a statistically significant positive correlation between the size of CAP lesion and the concentration of apoAI (R = 0.669; *p* = 0.012), and between TNF-α and hsCRP (R = 0.690, *p* = 0.018).

In patients over 50 years of age statistically significant correlations were found between: the size of CAP lesion and LpPLA2 level (R = 0.493, *p* = 0.023), and the size of CAP lesion and hsCRP level (R = 0.425, *p* = 0.043), and significant correlations between the concentration of TNF-α and hsCRP level (R = 0.624, *p* = 0.005), and between the concentration of TNF-α and IL-6 level (R = 0.26, *p* = 0.009). Moreover, between the concentration of LpPLA2 and TG level (R = 0.437, *p* = 0.032), that of LpPLA2 and TC level (R = 0.473, *p* = 0.032), that of LpPLA2 and LDL-C level (R = 0.518, *p* = 0.009), LpPLA2 and nonHDL-C (R = 0.573, *p* = 0.003), and between that of IL-6 and the age (0.597, *p* = 0.0015).

In patients with apoAI ≤ 150 mg/dL, statistically significant positive correlations was found between: the CAP lesion size and LpPLA2 level (R = 0.719, *p* = 0.0003) and the CAP lesion size and the age (R = 0.776, *p* = 0.0001) and between the CAP lesion size and TG level (R = 0.515, *p* = 0.011), and the CAP lesion size TG/HDL-C ratio (R = 0.414, *p* = 0.04).

However, in patients with apoAI ≥ 150 mg/dL level a negative correlation between the size of CAP lesion and LpPLA2 level (R = −0.727, *p* = 0.007) was found.

## Discussion

The literature data indicate how much incomplete are the studies on chronic infections in periodontal diseases, and particularly in chronic apical periodontitis. In recent years the concept of focal infection has changed, and mostly shows the correlation between chronic periodontal inflammation and systemic diseases [15, 16, 21–23]. The patients in our research were generally in good health condition without acute periodontal diseases and infections, caring for oral hygiene. It should be also noted that our patients did not take statins. Because the patient’s age varied (32–69 years) the size of CAP lesion were different. The results showed that the concentration of inflammatory markers (concentration of IL-6, TNF-α, hsCRP), and LpPLA2 and the size of CAP lesion were significantly the lowest in patients under 50 years of age, increased with age, and were the highest in relation to the control group, and patients ≤ 50 years of age as well as the oldest with largest CAP lesion size. These studies are consistent with the previous observations that the patient’s age has an impact on the healing process of chronic inflammation of apical teeth [24]. The studies of Starr et al. [25] showed that the adipocytes adipose tissue is the cause of elevated IL-6 and TNF-α levels, which may give rise to inflammation, tendency to infections, atherosclerosis and heart diseases.

A considerable differentiation of the concentration data of lipids, apoB, and apoAI indicates that there were patients with dyslipidaemia and dyslipoproteinaemia characterized by elevated levels of atherosclerotic lipid and lipoprotein profiles. Therefore, all patients were divided into groups with apoAI ≥ 150 mg/dL and apoAI < 150 mg/dL level. In patients with apoAI ≤ 150 mg/dL the IL-6, TNF-α, hsCRP and LpPLA2 concentration were significantly higher as compared to the patients with apoAI ≥ 150 mg/dL level and control group, whereas the size of CAP lesion was similar in both studied patient groups. The concentration of lipid and lipoprotein was still various, but it was worse in patients with apoA ≤ 150 mg/dL concentration. The results indicated that the increasing level of LpPLA2 and inflammatory markers and decreased apoAI concentration could cause systemic infections and diseases in all patients regardless of the age. These studies also suggested that dyslipidaemia and dyslipoproteinaemia depend on individual patient’s condition, his environmental and genetic predisposition to the occurrence of atherosclerosis, heart diseases, stroke, kidney diseases, which together with acquired dysfunctions with age pose a serious threat to the health and life of such patients [21]. In all patients we showed a strong positive correlation between the concentration of TNF-α and IL-6 level, and that of TNF-α and hsCRP level. IL-6 level correlated positively with hsCRP concentration and age. These results are consistent with the studies of other authors [26]. A positive relationship was demonstrated between the concentration of LpPLA2 and lipids (TG, TC, LDL-C and nonHDL-C) and that of LpPLA2 and lipid ratios (TC/HDL-C, LDL-C/HDL-C, TG/HDL-C) and between that of LpPLA2 and the patient’s age. These positive correlations indicated that together with increasing concentration of LpPLA2, the lipids (TG, TC, LDL-C and nonHDL-C) and atherogenic lipid and lipoprotein ratios also increased, that suggested a risk of atherosclerosis in these patients [27]. Moreover, in the group of patients over 50 years of age a positive correlation between the size of CAP lesion and hsCRP concentration and between the size of CAP lesion and LpPLA2 level was shown. The correlations indicated that the inflammation might increase the concentration of TC, TG, LDL-C, nonHDL-C and LpPLA2 and suggested that the inflammation might induce dyslipoproteinaemia and increase TG in triglyceride-rich lipoprotein and TC in cholesterol-rich lipoprotein, which could be effected by dysfunction of the vessel wall. These correlations may explain a high concentration of TC, nonHDL-C, TG, LDL-C and apoB in some patients with CAP [2, 11–13, 28]. Elevated Lp-PLA2 mass was related with endothelial dysfunction, carotid atherosclerosis, impaired coronary flow reserve and increased arterial stiffness and adverse outcome in CVD patients [12]. Plasma (Lp-PLA2) binds to low-density lipoprotein. The levels of Lp-PLA2 reflected the plaque burden, and are up-regulated in acute coronary syndrome (ACS). Lp-PLA2 levels were related to plaque stability and they might be a diagnostic biomarker for ACS [13]. In the group of patients with CAP under 50 years of age a positive correlation between the level of TNF-α and hsCRP concentration indicated increased activity of inflammatory markers. Moreover, our studies showed a positive correlation between the concentration of apoAI and the size of CAP lesion, and a negative correlation between the size of CAP lesion and LpPLA2 level. This positive correlation between the size of CAP lesion and apoAI level may suggest limiting CAP lesion size and the risk of systemic infections in young patients with high concentration of apoAI, and high antiinflammatory functions of apoAI and HDL particles [27–29]. A negative correlation between size of CAP lesion and LpPLA2 level may also suggest antiinflammatory function of LpPLA2, and limiting the size of CAP lesion and systemic risk of infections in patients with elevated concentration of apoAI, especially in younger people. We report for the first time that both LpPLA2 level and apoAI concentration can limit inflammation spread in patients with CAP. Several lines of evidence suggest that the role of plasma Lp-PLA2 in atherosclerosis may depend on the type of lipoprotein particle with which this enzyme is associated. The HDL-associated Lp-PLA2 may express anti-atherogenic activities and is also independently associated with a lower risk of cardiac death [27]. Thus, our studies suggest that the prognostic role of Lp-PLA2 in chronic CVD may be explained by a generalized detrimental effect of lipase on the endothelial function and arterial wall properties [12]. It appears that periodontitis induces proatherogenic lipoprotein patterns, and the association turns out to be the strongest for apoB-100-containing lipoproteins, but details about the potential mechanisms and the specific role of VLDL in this context have remained largely unknown [9, 30]. Rufail et al. [31] showed increased TG in cases of periodontitis as a result of increases in VLDL and a more robust increase in IDL particles, which appears to be more predictive of atherosclerosis progression than LDL, leading to increased cardiovascular (CV) risk in these subjects. Pussinen al. [32] indicated that periodontal infections may alter the antiatherogenic potency of HDL, indicating that HDL may effect the CV risk of subjects in ways that are not related to the concentration of these particles. The authors present that lipoproteins (especially HDL) are effective in binding and neutralizing lipopolysaccharide (LPS) of Gram-negative bacteria, limiting thereby the expression of cytokines and lipid peroxidation, and have an antioxidant feature. However, the protective role of HDL may be related not only to the level but also to the quality of HDL [9, 33]. Recently Petersen et al. [34] reported that CAP correlated positively with the aortic atherosclerotic burden. In the regression models, CAP without endodontic treatment was found to be an important factor, but not in apical radiolucencies of teeth with endodontic treatment.

## Conclusions

The findings suggest that in patients with CAP together with aging the size of CAP lesion and the concentration of inflammatory markers and LpPLA2 mass increase. The correlation between CAP lesion size and LpPLA2 and between CAP lesion size and TG level can cause increase TG in atherogenic apoB-containing triglyceride-rich lipoprotein and TC in cholesterol-rich lipoprotein. The patients with low levels of apoAI and high LpPLA2 can have a higher risk of odontogenic diseases, the progression of atherosclerosis and coronary heart diseases. Moreover, we have found a positive correlation between apoAI level and the CAP lesion size and a negative correlation between LpPLA2 level and the CAP lesion size. The results suggest for the first time that apoAI and LpPLA2 in HDL particles have antiinflammatory action and together can limit the CAP lesion size in patient with a higher apoAI level. The literature data on the distribution of lipoprotein particles in subjects are still insufficient, so this problem requires further investigations.
